# Mesenchymal stromal cells-extracellular vesicles: protein corona as a camouflage mechanism?

**DOI:** 10.20517/evcna.2024.78

**Published:** 2024-11-28

**Authors:** Enrico Ragni, Michela Taiana

**Affiliations:** ^1^Laboratorio di Biotecnologie Applicate all’Ortopedia, IRCCS Ospedale Galeazzi - Sant’Ambrogio, Milano 20157, Italy.

**Keywords:** Mesenchymal stromal cells, extracellular vesicles, protein corona

## Abstract

Mesenchymal stromal cells (MSCs) showed promising potential for regenerative and therapeutic applications for several pathologies and conditions. Their potential is mainly ascribed to the factors and extracellular vesicles (EVs) they release, which are now envisioned as cell-free therapeutics in cutting-edge clinical studies. A main cornerstone is the preferential uptake by target cells and tissues, in contrast to clearance by phagocytic cells or removal from circulation before reaching the final destination. Recent literature has suggested how the surface properties of EVs might influence their half-life, bio-distribution, and specific uptake. In particular, the concept of a protein corona surrounding EVs emerged. Especially for culture-purified EVs, the process of tailoring a treatment or tissue-specific corona was explored. Liam-Or *et al*. examined the impact of protein corona on MSC-EVs when specific proteins, preferentially albumin, were adsorbed from media on the EV surface before isolation. This resulted in improved uptake by liver parenchymal cells and reduced incorporation by macrophages, together with an increased half-life in the circulation system. Thus, producing MSC-EVs with an albumin-enriched protein corona might be a camouflage strategy to enhance non-phagocytic uptake in the liver. This research might be a milestone for future studies on other EVs-camouflage approaches tailored to specific tissues and therapeutic applications.

## MAIN TEXT

Mesenchymal stromal cells (MSCs) were first identified in the bone marrow in the late 1960s by Friedenstein *et al*. as multipotent cells able to differentiate into osteoblasts, adipocytes, and chondrocytes *in vitro*^[1]^. In recent years, it became clear that similar cell types might be identified in several other tissues^[2]^, including adipose tissue, placenta, umbilical cord, and umbilical cord blood. The first detailed characterization of MSCs was performed by Caplan^[3]^ in the 1990s, who proposed in the 2010s the nowadays most accepted concept of “Medicinal Signalling Cells”^[4]^, due to their ability to secrete bioactive factors (the secretome) that are immunomodulatory and trophic (regenerative)^[5]^. Therefore, in the clinical setting, MSCs are now believed to stimulate the patient’s own site-specific and tissue-specific resident stem cells, rather than differentiate into the damaged tissues; additionally, MSCs also modulate the immune response^[6]^. As of October 2024, almost 1,700 clinical trials are registered (https://clinicaltrials.gov/, search term: Mesenchymal Stem/Stromal Cells), evaluating the effectiveness of MSCs in treating, *inter alia*, orthopedic conditions, cardiovascular diseases, graft-versus-host disease (GvHD), and brain and neurological disorders, with some studies also focusing on immune system diseases.

Due to its properties resembling those of secreting cells, the interest in MSC-derived secretome is notably increasing, with nearly 30 registered clinical trials investigating its potential for osteoarthritis, cerebrovascular and neurological pathologies, and respiratory diseases, including COVID-19 (https://clinicaltrials.gov/, search term: Mesenchymal Stem/Stromal Cells Secretome). The secretome^[6]^ consists of both soluble factors (growth factors, cytokines, chemokines and hormones) and lipid nanoparticles like extracellular vesicles (EVs). EVs are classified into small EVs (sEVs, < 200 nm, also improperly called “exosomes”) or large EVs (lEVs, > 200 nm) depending on their size according to Minimal Information for EVs 2023^[7]^. Due to recent evidence that MSC-EVs have an important role in mediating the regenerative and immunomodulatory properties of MSCs^[8]^, purified EVs are also currently tested in almost 50 clinical studies (https://clinicaltrials.gov/, search term: Mesenchymal Stem/Stromal Cells EVs/Exosomes). EVs shuttle several molecules, such as proteins, lipids and nucleic acids, including miRNAs^[9]^. In particular, miRNAs were shown to be involved in regenerative and immune-related processes^[10]^, such as osteoarthritis^[11]^, neurological^[12]^ and cardiovascular diseases^[13]^, and the immune system^[14]^. Despite this increasing knowledge in EV cargo, an underestimated issue is the molecular characterization of how EVs interact with and are taken up by target cells and tissues. EVs binding is regulated by a complex process that involves several factors, including the composition of the membranes, such as protein, lipid, and glycan. As an example, tetraspanins in EVs play an essential role interacting with integrins and adhesion receptors^[15]^, as well as lipid membrane components, like phosphatidylserine, do with target cell membrane receptors^[16]^, and proteoglycans with lectins^[17]^.

Recently, together with proteins, lipids, and glycans that define the bilayer architecture of EVs, other molecules have been described as involved with EV interaction and targeting. A set of proteins and other biological compounds have been demonstrated to be adsorbed around EVs and constitute an external layer named corona^[18]^. Two distinct layers may be described: inner (hard) and outer (soft). Irreversible absorption of proteins around EVs leads to the formation of the inner hard layer, while reversible interaction of low-affinity proteins results in the formation of the outer soft layer^[19]^. Very little knowledge is present regarding the composition of soft and hard layers, although the formation of the corona around EVs inside aqueous phases was postulated to happen via electrostatic interactions and protein aggregation in an environment-dependent fashion^[20]^. As an example, for plasma EVs, there is a high overlap in the composition of their protein corona with plasma protein aggregates^[20]^ that may correlate with the surface of EVs. Accordingly, a corona layer was found on human-platelet-lysate-derived EVs, which exhibited regenerative and immunomodulatory properties on T cells *in vitro*^[21]^. For culture-derived EVs, such as MSC-EVs, a two-step process may occur. During production in cell culture, a first layer can be assembled, and after injection, the interaction with biological fluids may lead to the formation of an environment-dependent layer. Therefore, both a “general” layer dependent on the production process and a “specific” layer related to the injection route have to be studied for clinical translation. Consistently, a corona containing proangiogenic factors from placental expanded MSCs was found on their EVs, and this corona played a key role in mediating vascularized skin regeneration *in vivo*^[22]^. However, the relative contribution of these corona layers to particle properties and *in vivo* behavior has not been thoroughly investigated to date.

A recent study by Liam-Or *et al*. studied how culturing methods affect MSC-EVs corona compositions and impact accumulation in liver cells and how targeting is dependent on key proteins that mediate the *in vivo* behavior of EVs^[23]^. EVs were produced from umbilical cord MSCs (ucMSCs) in both 2-dimensional and 3-dimensional cultures. Serum-free basal medium and 20% KnockOut serum replacement (KO)-containing basal medium were used for 2D and 3D MSC cultures, respectively. EVs were purified with a sucrose gradient centrifugation before a final wash step and stored at -80 °C^[23]^. Although having a similar marker expression, zeta-potential values and size, EV_3D_ had a three-fold higher protein content than EV_2D_. Consistently, distinct protein profiles between EV_2D_ and EV_3D_ can be observed, with EV_3D_ showing similarities to KO medium proteins which were absent in EV_2D_^[23]^. This was confirmed by mass spectrometry, with serum albumin being among the most abundant proteins in both EV_3D_ and KO supplements, forming the inner corona during culture. On the contrary, EV_2D_ cultured in basal medium without supplements were covered with MSC secretome proteins^[23]^. Additionally, exposing EVs to EVs-depleted FBS (fetal bovine serum) allowed for the formation of an outer corona with different preferential compositions when comparing 2D and 3D (EV with bound hard corona: HC-EV_2D_ and HC-EV_3D_). For example, bovine serum albumin was again more abundant in EV_3D_ with respect to EV_2D_. Thus, culture medium (pure basal or KO-supplemented) and subsequent FBS incubation allowed for the formation of specific layers, termed the primary and secondary corona, respectively^[23]^.

The authors further explored the role of different corona in EV uptake using both *in vitro* and *in vivo* systems. In the *in vitro* study, they characterized the uptake of EVs by macrophages and liver parenchymal cells, focusing on strategies to enhance EV accumulation in liver cells other than in phagocytic cells. The presence of the corona increased the uptake in both cell types for EV_2D_, while EV_3D_ exhibited a significant reduction in uptake by phagocytic cells and a corresponding increase in uptake by parenchymal cells^[23]^. Overall, 3D-cultured EVs were preferentially internalized by parenchymal cells, and their reduced uptake by phagocytic cells indicated a more favorable interaction with the liver microenvironment *in vitro*. These results were confirmed *in vivo* through a biodistribution study in mice. Both EV types recorded the highest accumulation in the spleen and liver, followed by the lungs and kidneys. Notably, EV_3D_ displayed a stronger signal in the liver, while EV_2D_ was detected at higher levels in urine, suggesting a shorter half-life for EV_2D_^[23]^. Further analysis dissected the uptake in liver subpopulations. No preferential uptake was observed between EV_2D_ and EV_3D_ for Kupffer cells. However, EV_3D_ demonstrated higher uptake in hepatocytes, endothelial cells, and stellate cells^[23]^. This indicates that while macrophages do not discriminate between EV types, liver parenchymal cells favor EV_3D_. These characteristics, along with the faster clearance of EV_2D_, were further confirmed by bioinformatics analyses of EVs and their associated corona proteins. Specifically, EV_2D_ is enriched with immunoglobulin heavy constant mu, a protein linked to phagocyte-mediated engulfment and complement activation. Additionally, the corona of EV2D contained proteins that promote EV clearance^[23]^. These findings suggest that EV_2D_ clearance might be promoted by opsonization, complement activation, and subsequent phagocytosis.

Eventually, authors proposed that albumin on the inner corona may attract more albumin to form the outer layer, and therefore, increased uptake observed for albumin-coated EV_3D_ might be dependent on albumin receptors and their subtype on target cells and tissues. The first hypothesis was confirmed by producing EV_2D_ in the presence of exogenous albumin, which enabled the formation of an albumin-enriched inner layer that further attracted albumin^[23]^ for the second layer. The second hypothesis was endorsed in experiments in which blocking albumin receptors reduced liver uptake of both EV_3D_ and EV_2D_ produced in the presence of albumin. Of note, the characteristics of EV_2D_ were observed only when vesicles were generated in the presence of albumin, not when particles were supplemented after their isolation^[23]^. This implies that the formation of the first corona at the site of secretion is essential.

These results showed how albumin coating is a requisite for the efficient uptake of EVs by hepatic cells and how it may prolong the half-life of EVs. Supporting this, a recent study demonstrated that an albumin-dominated corona formed on lipid-like nanoparticles preferentially binds to receptors expressed on primary hepatocytes^[24]^. Additionally, EVs engineered to bind albumin exhibit extended circulation times, resulting in prolonged half-life^[25]^. Further research is needed to understand the possibility to prolong the blood-circulation time of albumin-enriched corona on EVs, a crucial requisite for prolonged therapeutic activity that may also depend on other key factors.

In this frame, the choice between 2D and 3D cell culture is a crucial step. In 2D cultures, cells grow on flat surfaces, often leading to altered cell morphology and less natural protein expression. This artificial environment may produce EVs with atypical protein coronas that do not accurately represent *in vivo* conditions, potentially limiting their camouflage efficacy in biological systems^[26]^. In contrast, 3D cultures better mimic *in vivo* environments by facilitating complex cell-cell and cell-matrix interactions, leading to more physiologically relevant EVs with protein coronas closer to those found in natural tissue. These EVs may display enhanced immune evasion and targeted delivery capabilities^[27]^. However, 3D cultures present challenges, including higher complexity, longer culture times, and increased cost. Additionally, the heterogeneity within 3D systems can make EV characterization difficult, as it may result in diverse EV subpopulations with varying protein coronas^[28]^. Ultimately, while 3D cultures offer a more accurate reflection of *in vivo* EV protein composition and camouflage potential, they are more demanding to manage and analyze.

Overall, the “protein corona-extracellular vesicle complex” represents a promising approach in EV-based therapeutic delivery, particularly for improved cell targeting and immune evasion. The protein corona significantly alters the interactions of EVs with cells and immune components. Understanding and manipulating this complex offers exciting opportunities but also poses several challenges. A key challenge is the lack of control over the composition of the protein corona, which can vary depending on the individual’s physiology and disease state, thus impacting targeting efficiency and immune evasion. Additionally, protein corona formation can mask EV surface proteins critical for binding to target cells, reducing therapeutic specificity. Further research is needed to develop methods for controlling corona composition, perhaps by engineering EV surfaces to selectively recruit beneficial proteins for immune evasion and cellular targeting^[18]^. Moreover, developing standard protocols for corona-EV complex characterization is essential for clinical translation, as these parameters affect biodistribution and therapeutic efficacy^[29]^. As insights into corona-EV interactions deepen, synthetic corona layers and tailored EV engineering may enable more precise, patient-specific treatments with fewer immunogenic risks.

In conclusion, Liam-Or *et al*. confirmed the contribution of corona enriched with albumin in prolonging the half-life of EVs and enhancing their uptake in liver parenchymal cells, particularly in relation to macrophages^[23]^. Future research will be crucial to tailor EV features for clinical applications, particularly those derived from MSCs, which have shown promise as therapeutic candidates for various diseases and pathologies.

## DECLARATIONS

### Availability of data and materials

Raw data are available at: https://osf.io/pd9tb/?view_only=27ca5142990444aeb903eb86fe5748e1.

### Copyright

© The Authors 2024.
